# In Vitro Evaluation of the Antifungal Properties of *Bixa orellana* L. Essential Oil from the Ecuadorian Amazon Against *Candida albicans* (ATCC 10231)

**DOI:** 10.3390/life14121628

**Published:** 2024-12-09

**Authors:** María Belén Cruz Berrú, María Coraima Mora García, Sandra Luisa Soria Re, Jannys Lizeth Rivera Barreto, Luis Ramón Bravo Sánchez, Matteo Radice, Stefano Manfredini, Reinier Abreu-Naranjo

**Affiliations:** 1 Carrera de Biología, Facultad de Ciencias de la Vida, Universidad Estatal Amazónica (UEA), Vía Tena km 2½, Puyo 160150, Pastaza, Ecuador; maria.belen77@hotmail.com (M.B.C.B.); coraima.mora.g@gmail.com (M.C.M.G.); 2 Facultad de Ciencias de la Tierra, Universidad Estatal Amazónica (UEA), Vía Tena km 2½, Puyo 160150, Pastaza, Ecuador; ssoria@uea.edu.ec (S.L.S.R.); jl.riverab@uea.edu.ec (J.L.R.B.); lbravo@uea.edu.ec (L.R.B.S.); mradice@uea.edu.ec (M.R.); rabreu@uea.edu.ec (R.A.-N.); 3 Department of Life Sciences and Biotechnology, University of Ferrara, 44121 Ferrara, Italy

**Keywords:** anti-candidiasis activity, sesquiterpenes, biodiversity, natural products, dihydroedulan

## Abstract

Essential oils are investigated due to their biological activity, and the Amazon rainforest, with its rich biodiversity, is a promising source of therapeutic compounds. The aim of this study was to evaluate the essential oil from the leaves of *Bixa orellana* as an antifungal agent, thus contributing to the search for alternatives that can address the growing resistance to conventional antifungals. *B. orellana* leaves were collected in the Ecuadorian Amazon and their essential oil was obtained by steam distillation. Their chemical composition was analysed by Gas Chromatography-Mass Spectrometry (GC-MS) and their antifungal activity against *Candida albicans* was evaluated using the Kirby–Bauer disc diffusion method (ATCC 10231), with nystatin as a positive control. GC-MS analysis revealed the presence of 60 compounds, the main ones being dihydroedulan (27.5%), β-caryophyllene (10.3%), nerolidol (7.21%), trans-β-bergamotene (5.73%), α-santalene (4.94%) and trans-α-bergamotene (4.26%). The essential oil showed moderate antifungal activity against *C. albicans*, producing an inhibition halo of 13 mm in diameter, which is 48% of the inhibition observed with nystatin (27 mm). The presence of sesquiterpenes, such as β-caryophyllene, known for its membrane-disrupting properties, probably contributes to the observed antifungal effects. The study highlights the potential of *B. orellana* essential oil as a natural antifungal agent; however, further research is required to evaluate its efficacy against a wider range of pathogenic fungi, its possible synergistic effects with conventional antifungals and its safety and efficacy in vivo.

## 1. Introduction

*Candida albicans* is a fungal pathogen that causes a variety of infections, mainly affecting immunocompromised individuals. The fungus is responsible for both superficial infections, such as oral and vaginal candidiasis and more serious systemic infections [1]. One of the main challenges in treating these infections is their increasing resistance to conventional antifungals, such as nystatin and fluconazole. 

In the case of *C. albicans*, the main mechanisms of resistance to antifungal drugs have been elucidated as genetic mutations encoding certain biosynthetic pathways and the overexpression of the efflux pump after exposure to antifungal drugs. These resistance mechanisms are present in the planktonic form as well as in the structure called a biofilm. In addition, the extracellular matrix of the biofilm has a high density of cells, persister cells and sterols. The structure acts as a physical barrier that prevents the penetration of drugs by making the pathogen inaccessible [2,3]. This situation calls for research into alternative therapies, such as EOs with antifungal properties, which can complement available treatments and help to reduce the emergence of resistance [4,5].

Essential oils (EOs) are volatile compounds obtained from various parts of plant matrices, such as flowers, leaves, bark, roots and fruit, using methods like hydrodistillation, steam distillation or solvent extraction. These oils consist of a variety of low-molecular-weight chemical constituents, including alcohols, polyphenols, terpenoids, carbonyls and aliphatic compounds, which contribute to their fragrance and biological properties [6]. Due to their therapeutic and aromatic properties, EOs are widely used in the health, cosmetic and pharmaceutic industries. Furthermore, in recent decades, interest in these oils has increased significantly due to their proven antimicrobial, antifungal and antioxidant activities, positioning them as promising alternatives to conventional synthetic treatments [7,8]. These properties are mainly related to their ability to prevent infections and protect against oxidative stress [9,10]. 

The antifungal activity of essential oils has been widely documented and is one of the many biological properties for which they are studied, such as the antibacterial activity, the antiproliferative effect and other potential medicinal applications [11,12,13]. The mechanism of action of essential oils mainly involves damaging cell walls and cell membrane structures, or increasing their permeability, and inhibiting mitochondrial activities. These effects limit fungal proliferation and mycotoxin production [14]. The potential application of EOs in the treatment of *C. albicans* infections has also been extensively studied, showing promising preliminary data, either during tests of synergic activity with conventional antifungals or the inhibition of biofilm formation [15,16].

In this context, the Ecuadorian Amazon, one of the most biodiverse regions in the world, has a great potential for providing essential oils with bioactive properties, which are still largely unexplored. Around 10% of the world’s known plant species are found in the Ecuadorian Amazon [17]. This remarkable biological wealth includes a wide variety of plants with a history of medicinal use in local cultures. For generations, medicinal plants have been used by various ethnic groups to treat a wide range of ailments [18]. However, many have not yet been studied in depth, especially with regard to their bioactive properties and therapeutic uses. Research on these compounds is essential in order to exploit the medicinal potential of both well-known and less-studied plants [19]. Among the species of interest, *B. orellana* has been recognised for its traditional applications and bioactive composition.

*B. Orellana* (local common name: achiote) is a plant native to Latin America that has been traditionally used both for its medicinal properties and for its value as a natural dye [20,21]. In Indigenous cultures, it has been used to treat a variety of ailments, such as skin infections, digestive problems and fever, as well as in spiritual rituals [22]. Its best-known applications are as a source of pigment, food, cosmetic and textile dye due to the high content of bixin in its seeds [23]. These uses, combined with its therapeutic properties, have positioned it as a plant of interest for both traditional medicine and industrial applications.

In addition to its traditional relevance, recent research has revealed that *B. orellana* EO contains a number of bioactive compounds, including sesquiterpenes and monoterpenes [24]. Compounds such as β-caryophyllene and trans-β-bergamotene, present in its EO, have been shown to possess antimicrobial and anti-inflammatory properties [25,26]. These findings suggest that annatto EO may have an antifungal potential that has not yet been fully investigated, positioning it as a source of interest for the development of natural therapeutic agents against fungal infections.

In this regard, *B. orellana* essential oil shows therapeutic potential as a natural alternative to conventional antifungals. However, there is a lack of specific studies on its antifungal activity, particularly against *C. albicans*, highlighting the need to explore its properties in this context. The purpose of the present study was to evaluate the essential oil of *B. orellana* leaves as an antifungal agent, thus contributing to the search for alternatives that can address the growing resistance to conventional antifungals.

## 2. Materials and Methods

### 2.1. Collection and Preparation of Plant Material

**Figure 1:**
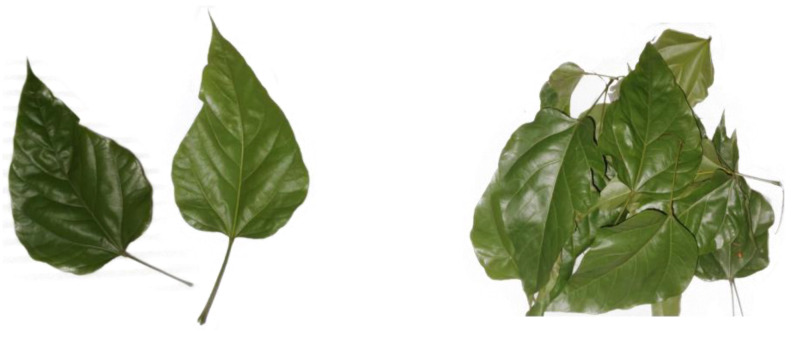
*B. orellana* leaves.

The leaves of *B. orellana* (Figure 1) were acquired at the ‘Mercado del Centro Agrícola’ in the city of Puyo, Pastaza province, in September 2023. Botanical identification was carried out by Dr. Diego Gutiérrez del Pozo at the Herbarium of the Universidad Estatal Amazónica (ECUAMZ), Puyo, Ecuador. The leaves came from the community of Canelos, canton Pastaza, Pastaza province, Ecuador. Subsequently, leaves in good condition and free of damage or signs of disease were selected and washed with distilled water to remove any residues or contaminants. Then, they were dried on filter paper at room temperature (20–25 °C) in accordance with Crespo et al. [27]. Once dry, the leaves were ground using a mill (Model: 4, Thomas Wiley Mill Co., Swedesboro, NJ, USA), achieving an average particle size of approximately 1.0 mm. The ground material was then placed in hermetically sealed polyethylene bags until analysis [28].

### 2.2. EO Extraction from B. orellana L. by Steam Distillation

The essential oil from the leaves of *B. orellana* was obtained using the steam distillation method. Each dried and ground plant sample was placed in a FIGMAY laboratory-scale essential oil extractor (model: FIGMAY S.R.L. laboratory scale, Córdoba, Argentina), according to the method described by Crespo et al. [27]. The distillation was carried out with a continuous flow of steam until successive readings of the oil volume remained constant. Multiple runs were performed to obtain a sufficient quantity of essential oil. Subsequently, the oil was treated with anhydrous sodium sulfate to remove residual moisture, then filtered and stored at 4 °C in sealed vials, protected from light. 

### 2.3. Gas Chromatography-Mass Spectrometry (GC-MS) Analysis

*B. orellana* leaf EO was analysed by GC-MS using a Shimadzu model QP2020 NX (Shimadzu Europa, Duisburg, Germany) equipped with a split/splitless injector and an AOC-20i autosampler; the fused silica capillary column was a 30 m × 0.25 mm I.D. × 0.25 μm Rtx—5 MS (Merck KGaA, Darmstadt, Germany), according to the method described by Valarezo et al. [24], with slight modifications. For the GC-MS analysis, 0.5 mL of *B. orellana* EO was mixed with 4.5 mL of analytical-grade hexane and a volume of 1 µL was injected into the system. The GC oven temperature was programmed as follows: from 55 to 100 °C at a heating rate of 1 °C/min, then increased at a rate of 5 °C/min up to 250 °C. Details were as follows—carrier gas: helium (99.99%); mobile phase flow rate: 1.10 mL/min; linear velocity: 40 cm/s; purge flow: 3.00 mL/min; split ratio: 20.0; and stationary phase: 5% diphenyl/95% dimethylpolysiloxane (low polarity/low bleed). The GC-MS chromatograms were obtained and analysed with the GC-MS Solution software version 4.50 (Shimadzu Europa, Duisburg, Germany). The peaks were identified using the FFNSC 4.0 mass spectral library (Shimadzu Europa, Duisburg, Germany), applying a mass spectral similarity threshold of 80% or higher.

### 2.4. Fungal Strain and Culture Conditions

The microorganism *C. albicans* (ATCC 10231) was used to evaluate the antifungal activity of the EO from *B. orellana* leaves. The strain was purchased from Medibac Laboratories (Guayaquil, Ecuador) and stored at −80 °C in the Microbiology Laboratory of the Universidad Estatal Amazónica until use.

First, the *C. albicans* ATCC 10231 strain was reactivated by incubating it in sealed test tubes containing potato dextrose agar (PDA, BD Bioxon^®^, Cuautitlán Izcalli, Mexico) prepared at a concentration of 39 g/L in water at 30 °C for 48 h, following standard microbiological procedures [29]. The strain was subsequently diluted in Sabouraud’s dextrose broth at a concentration of 30 g/L until a suspension with a turbidity of 0.5 on the McFarland scale was achieved, which corresponds to a concentration of 1.5 × 10⁸ CFU/mL [29]. The McFarland standard was prepared by mixing 24.875 mL of 1% BaCl_2_ with 0.125 mL of 1% H_2_SO_4_. The absorbance of both the McFarland standard and the microbial suspension was measured at 625 nm using a Lambda 25 UV/VIS spectrophotometer (Perkin Elmer, Waltham, MA, USA) until the microbial suspension concentration matched the McFarland 0.5 standard.

### 2.5. In Vitro Evaluation of the Inhibitory Effect of the B. orellana EO

The antifungal activity of *B. orellana* EO against *Candida albicans* (ATCC 10231) was evaluated using the Kirby–Bauer method [30]. Sterile filter paper discs of 5 mm diameter, impregnated with 20 μL of the essential oil, were carefully placed at equal distances from the edge of Petri dishes containing PDA previously inoculated with the *C. albicans* strain. Nystatin (100,000 UL/mL, 60 mL suspension) was used as a positive control. The plates were sealed with Parafilm^®^ to preserve the integrity of the medium and incubated at 37 °C for 48 h. The diameters of the complete inhibition zone were measured in millimetres, passing through the centre of each disc. Measurements were performed in triplicate for each disc using a digital vernier (Mitutoyo, Kawasaki, Japan), and the values of the measurements were averaged. The experiment was performed in triplicate to ensure the reproducibility of the results. 

## 3. Results

### 3.1. Extraction Yield of EO from B. orellana

The extraction of EO from *B. orellana* leaves was carried out using the steam distillation method. The amount of EO obtained was approximately 13 mL, yielding 0.08 mL/100 g, with a light-yellow colour and an intense aroma. This yield represents the amount of essential oil obtained in relation to the quantity of plant material used in the extraction process with a moisture content of 27.8%. 

### 3.2. Chemical Composition of the B. orellana EO

**Figure 2:**
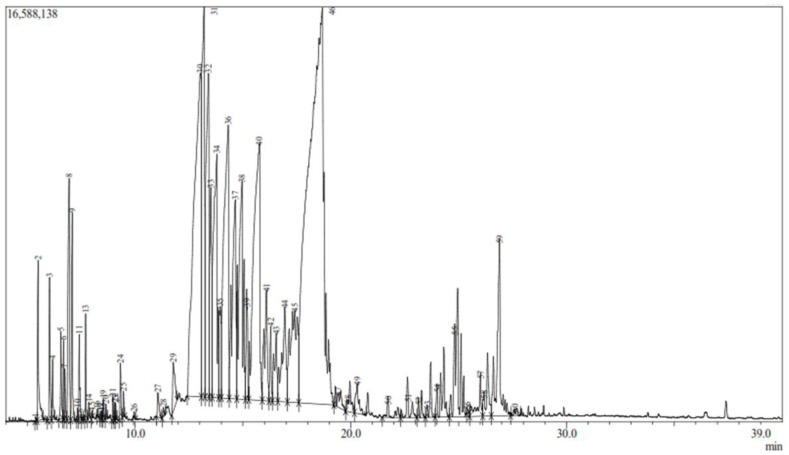
Chromatogram of the EO of *B. orellana*.

The GC-MS analysis of the EO extracted from *B. orellana* L. leaves revealed a total of 60 compounds, accounting for approximately 99.99% of the total chromatogram area (Figure 2). Compound identification was based on retention times and mass spectra, which were compared against the FFNSC 4.0 mass spectral library (Supplementary Materials). This analysis underscores the complex chemical profile of the essential oil.

For a more detailed and relevant analysis, only those compounds that exceeded 1% of the total chromatogram area were considered, as detailed in Table 1. Among the main components by relative abundance, dihydroedulan (27.5%), nerolidol (7.21%), trans-β-bergamotene (5.73%), α-santalene (4.94%), β-santalene (4.19%) and trans-α-bergamotene (4.26%) were prominent. 

The identified compounds are classified into chemical groups, such as sesquiterpenes, monoterpenes and diterpenes. Sesquiterpenes are the most represented group, featuring compounds like β-caryophyllene, α-santalene, β-santalene and nerolidol, noted for their high percentages. Other minor groups include diterpenes, such as phytol, along with some alicyclic and aromatic compounds.

### 3.3. Antifungal Activity

**Figure 3:**
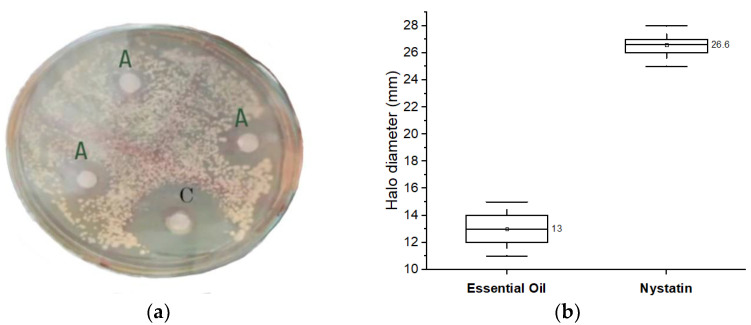
Antifungal activity of *B. orellana* EO: inhibition halos (**a**) and statistical comparison with nystatin (**b**).

The inhibitory capacity of *B. orellana* essential oil against *C. albicans* was evaluated by means of the Kirby–Bauer disc diffusion method. Figure 3 presents the results, compared with those obtained for nystatin, a standard antifungal used as a positive control.

In Figure 3a, the inhibition halos produced by *B. orellana* EO (A) and nystatin (C) are shown, while the quantitative analysis of the inhibition halos is presented in Figure 3b. Nystatin produced an inhibition halo of ~27 mm in diameter. According to the classification proposed by Morales et al. [31], this result falls within the high activity category (21–30 mm). Meanwhile, the essential oil of *B. orellana* generated a halo with an average diameter of 13 mm, which is classified as moderate activity (11–20 mm) on the same scale. These results indicate that *B. orellana* essential oil exhibits antifungal activity against *C. albicans*, reaching 48% of the inhibition observed with nystatin, an antifungal that has demonstrated effective activity in this type of assay [32].

## 4. Discussion

### 4.1. Extraction Yield of EO from B. orellana

Concerning the extraction yield of EO from *B. orellana*, the value obtained in this study is lower than that reported by Valarezo et al. [24], who obtained a yield of 0.13% by hydrodistillation from *B. orellana* leaves collected in El Dorado parish, Francisco de Orellana canton, Orellana Province, Ecuador. These differences in yield can be attributed to several factors, such as the variety, the part of the plant used, climatic conditions and the specific extraction process conditions. In this case, evidence from previous studies has pointed to a higher yield when oils are extracted by hydrodistillation compared to that obtained by the vapor entrainment method [33].

In addition, the drying temperature of the leaves also plays an important role in the yield of the essential oil obtained. A study by Oliveira Everton et al. [34] showed that drying temperature significantly affects both the quantity and quality of the essential oil extracted from *B. orellana* leaves. In particular, they found that drying at 45 °C produced the highest essential oil yield (2.23%), outperforming lower or higher temperatures, such as 35 °C (0.21%) and 55 °C (0.38%). This behaviour is attributed to the preservation of cell integrity at intermediate temperatures, which facilitates the extraction of the essential oil without causing a significant loss of volatile components. Therefore, it is possible that the lower yield obtained in our study is also due to the drying temperature of the leaves used before extracting the EO.

### 4.2. Chemical Composition of the B. orellana EO

Regarding the chemical composition of the *B. Orellana* EO, similar results were obtained by Giorgi et al. [35], who also reported that sesquiterpenes and monoterpenes are the main groups of volatile compounds present in the essential oil extracted from different parts of *B. orellana*, collected in the Upper Guamá River Reserve in the state of Pará in Brazil’s Amazon region. In their study, they identified β-caryophyllene, γ-caryophyllene, α-caryophyllene, α-copaene and D-germacrene as the major compounds. However, the difference between their results and the compounds identified in the present study can be attributed to a number of factors, including the environmental, geographical and growing conditions of plants, as well as possible variations in the time of collection of the plant material analysed. It is well documented in the literature that the chemical composition of essential oils can vary significantly depending on these extrinsic factors [36].

The dominant presence of sesquiterpenes in *B. orellana* essential oil suggests that it may possess diverse bioactive properties [37]. Sesquiterpenes, such as costunolide, have demonstrated remarkable in vitro antifungal activity against various pathogens, indicating that these compounds could contribute significantly to the antimicrobial properties of the essential oil [38]. Previous studies have also reported that certain sesquiterpenes exhibit anti-inflammatory and antioxidant properties, indicating that they are potential candidates for the development of natural therapeutic agents [39]. The ability of these compounds to modulate diverse biochemical pathways, such as inhibiting cyclooxygenase-2 (COX-2) and neutralising reactive oxygen species (ROS), has been documented in both cellular and animal models [37,40]. These findings underscore the importance of sesquiterpenes in both traditional medicine and modern pharmacological research. However, while the presence of these compounds in *B. orellana* essential oil is promising, further studies specifically focusing on this oil are necessary to determine its potential therapeutic applications. Such research would help elucidate the bioavailability and potential synergistic effects of the various compounds present in the oil.

Monoterpenes typically constitute up to 90% of essential oils and are known for their antimicrobial and antioxidant activities [40]. As mentioned above, dihydroedulan, a cyclic monoterpene, was identified as the most abundant compound in the essential oil of *B. orellana* in the present study.

Various investigations into monoterpenes have demonstrated their bioactive properties, including antimicrobial, antioxidant, anti-inflammatory and neuroprotective effects [41,42,43]. Compounds like α-pinene and limonene show antimicrobial activity, while 1,8-cineole demonstrates antioxidant properties by protecting cells from oxidative stress-induced apoptosis [43]. Additionally, some monoterpenes, such as β-caryophyllene, display neuroprotective potential by inducing antioxidant enzyme expression through transcription factors like Nrf2 [41]. These properties highlight the therapeutic potential of monoterpenes in various applications, from food preservation to pharmaceutical formulations. In this regard, the significant presence of dihydroedulan in *B. orellana* essential oil suggests that it could be an important contributor to the overall bioactivity of the oil.

### 4.3. Antifungal Activity

Finally, the antifungal activity observed in *B. orellana* EO could be explained by the presence of compounds with known bioactive properties. Very few studies exist on the biological activity of dihydroedulan; Naija et al. [44] mention the molecule’s antioxidant and antimicrobial properties. Concerning nerolidol, the literature reports preliminary results for neurodegenerative diseases and a common use as a cosmetic, detergent and food flavouring agent [45,46]. Although the specific bioactive properties of dihydroedulan and trans-β-bergamotene are less well documented compared to those of nerolidol and other terpenes, their prevalence suggests they may contribute significantly to the overall bioactivity of oils. β-Caryophyllene (10.3%), one of the major components identified, has been widely investigated for its antifungal effects against various pathogenic fungi [47]. Indeed, it is a major sesquiterpene in many essential oils and has been shown to have the ability to interact with fungal cell membrane lipids, compromising their structural integrity and, consequently, their viability [41]. This effect is due, in part, to the ability of terpenoids to increase membrane permeability, which can lead to cell lysis. Furthermore, previous studies have suggested that β-caryophyllene can induce the production of reactive oxygen species (ROS), resulting in additional oxidative damage to the fungal cell. The cyclic structure of certain sesquiterpenes, such as α-santalene, confers them greater stability, favouring their interaction with membrane lipid components, which could enhance their antifungal activity by inducing both structural damage and oxidative stress [48].

Compounds such as trans-β-bergamotene (5.73%) and α-santalene (4.94%) have also shown antifungal activity in other investigations [49,50,51]. Trans-bergamotene was also identified as an important compound of *Isolona dewevrei* essential oil, which presented preliminary in vitro anti-inflammatory potential [26]. Therefore, it is possible that the abundant presence of these sesquiterpene hydrocarbons contributes to the antifungal capacity of *B. orellana* L. essential oil, although further studies are required to establish direct correlations.

Whilst *B. orellana* essential oil does not reach the same level of inhibition as nystatin, it exhibits moderate antifungal activity against *C. albicans*, which may indicate its potential as a natural antifungal agent. However, an additional aspect to consider is the potential synergy between the components of *B. orellana* essential oil and conventional antifungal agents. Studies, such as that of Sempere-Ferre et al. [52], have demonstrated that combining essential oils with antifungals can enhance their activity and reduce the likelihood of resistance development in microorganisms. Currently, the authors found no studies of synergistic activity with conventional antifungals related to *B. orellana* oil or any of the following main components: dihydroedulan, trans-β-bergamotene, α-santalene, β-santalene and trans-α-bergamotene. The only data found concern the synergistic action of nerolidol and griseofulvin for *Trichophyton* spp. For all other compounds and for *B. orellana* essential oil, further research would be appropriate [53].

In this regard, recent studies have shown that essential oils such as *Mentha x piperita*, *Pelargonium graveolens* and *Melaleuca alternifolia* possess antifungal properties that demonstrate a strong synergy when combined with conventional antifungals. In their study on the combination of essential oils with diclofenac, Rosato et al. [54] showed that there is a significant decrease in the minimum inhibitory concentration (MIC) required to inhibit the growth of various strains of *Candida* spp. The essential oil of *M. piperita* showed a fractional interaction index (FICI) of 0.22, indicating a strong synergy, reducing the concentration of diclofenac from 2.05 to 0.06 μg/mL in the presence of the oil. This suggests that *B. orellana* essential oil, due to its sesquiterpene-rich composition, could have a similar synergistic effect, which could increase its antifungal efficacy.

## 5. Conclusions

This study provides valuable insights into the chemical composition and antifungal activity of *B. orellana* EO from the Ecuadorian Amazon. The results show a complex terpenoid profile with dihydroedulan (27.5%), β-caryophyllene (10.3%) and trans-β-bergamotene (4.26%) as major components. These compounds have previously been associated with significant bioactive properties, suggesting potential therapeutic applications. The EO showed moderate antifungal activity against *C. albicans*, achieving 48% of the inhibition observed with nystatin. This result positions *B. orellana* EO as a promising source of natural antifungal compounds. However, further studies are needed to investigate the antifungal activity against a wider range of pathogenic fungi, potential synergistic effects with conventional antifungal agents and the in vivo efficacy and safety. This research establishes *B. orellana* EO from the Ecuadorian Amazon as a promising source of bioactive compounds with potential applications in the development of natural antifungal agents and other therapeutic uses.

## Figures and Tables

**Table 1:** Chemical composition of the EO of *B. orellana* leaves.

RT (min)	(%)	Component	Molecular Weight (g/mol)
6.94	1.65	Cis-Ocimene	120.2
13.020	10.3	β-Caryophyllene	204.4
13.198	4.94	α-Santalene	204.4
13.404	4.26	Trans-α-Bergamotene	204.4
13.509	1.91	Bicyclo[2.2.1]heptane	204.4
13.790	4.19	β-Santalene	204.4
14.319	5.73	Trans-β-Bergamotene	204.4
14.653	3.51	β-Curcumene	202.4
14.954	4.13	3-(1,5-Dimethyl-4-hexenyl)-6-methylene-cyclohexene [S-(R*S*)]	204.4
15.756	7.21	Nerolidol	222.4
16.097	1.54	β-Caryophyllene oxide	220.4
16.945	1.74	β-Acorenol	222.4
17.408	3.14	Dehydrosesquicineole	218.4
18.659	27.5	Dihydroedulan	220.4
24.829	2.39	Biflora-4(10),15-diene	272.5
26.903	2.46	Phytol	296.5

## Data Availability

Complete data regarding essential oil composition are available in Supplementary Materials.

## Supplementary Materials

The following supporting information can be downloaded at: https://www.mdpi.com/article/10.3390/life14121628/s1, Table S1: Chemical composition of the essential oil of the leaves of *B. orellana* L.
