# Supplementary material for: In Vitro Evaluation of the Antifungal Properties of Bixa orellana L. Essential Oil from the Ecuadorian Amazon Against Candida albicans (ATCC 10231)
Source: Life (Basel). 2024 Dec 9;14(12):1628. doi: 10.3390/life14121628 (PMC11677816; doi:10.3390/life14121628)
Supplement: Supplementary file 1 [file life-14-01628-s001.zip › life-3316035-supplementary.pdf]

Supplementary Material 1

In Vitro Evaluation of the Antifungal Properties of Bixa orellana L. Essential Oil from the Ecuadorian Amazon against Candida albicans (ATCC 10231)

María Belén Cruz Berrú[1], María Coraima Mora García[1], Sandra Luisa Soria Re[2], Jannys Lizeth Rivera Barreto[2], Luis Ramón Bravo Sánchez[2], Matteo Radice[2], Stefano Manfredini[3]* and Reinier Abreu-Naranjo[2]

[1] Carrera de Biología, Facultad de Ciencias de la Vida, Universidad Estatal Amazónica (UEA), Vía Tena km 2½, Puyo, Pastaza, Ecuador. M.B.C.B maria.belen77@hotmail.com; M.C.M. G coraima.mora.g@gmail.com

[2] Facultad de Ciencias de la Tierra, Universidad Estatal Amazónica (UEA), Vía Tena km 2½, Puyo, Pastaza, Ecuador. S.L.S.R ssoria@uea.edu.ec; L.R.B. jl.riverab@uea.edu.ec; L.R.B.S lbravo@uea.edu.ec; R.A.N. rabreu@uea.edu.ec

3 Department of Life Sciences and Biotechnology, University of Ferrara, 44121 Ferrara, Italy

* Correspondence author: smanfred@unife.it

Table S1. Chemical composition of the essential oil of the leaves of *B. orellana* L.

Order	RT (min)	(%)	Component	Molecular weight (g/mol)
1	5,380	0,02	α-Thujene	136,2
2	5,500	0,79	Camphene	136,2
3	6,033	0,60	β-Pinene	136,2
4	6,171	0,23	β-Myrcene	136,2
5	6,561	0,37	o- Cymene	134,2
6	6,686	0,26	Limonene	136,2
7	6,742	0,22	α-Pinene	136,2
8	6,940	1,65	cis-ocimene	120,2
9	7,083	0,97	γ-Terpinene	136,2

10	7,327	0,03	p-Cimene	132,2
11	7,409	0,34	4-Isopropylidene-1-cyclohexene	108,2
12	7,560	0,01	3,5-Dimethylhexanoic acid	144,2
13	7,706	0,32	E-4,8-Dimethylnona-1,3,7-triene	138,3
14	7,850	0,05	E,Z-2,6-Dimethylocta-2,4,6-triene	120,2
15	8,001	0,02	Citronellal	154,3
16	8,246	0,02	Borneol	154,3
17	8,396	0,02	Terpinen-4-ol	154,3
18	8,476	0,02	Methyl salicylate	152,2
19	8,522	0,08	α-Terpineol	154,3
20	8,649	0,06	2,6-Dimethylocta-3,5,7-trien-2-ol	154,3
21	8,969	0,08	Citronellol	156,3
22	9,064	0,05	Neral	154,3
23	9,100	0,05	Norcaradiene	136,2
24	9,322	0,22	Geraniol	154,3
25	9,462	0,09	Citral	152,2
26	9,958	0,02	Cipren	204,4
27	11,056	0,19	γ-Elemene	204,4
28	11,308	0,05	α-Cubebene	204,4
29	11,771	0,50	Bicyclogermacrene	204,4
30	13,020	10,3	β-Caryophyllene	204,4
31	13,198	4,94	α-Santalene	204,4
32	13,404	4,26	Trans-α-Bergamotene	204,4
33	13,509	1,91	Bicyclo[2.2.1]heptane	204,4
34	13,790	4,19	β-Santalene	204,4
35	13,951	0,91	1-Methyl-4-(6-methylhept-5-en-2-yl)cyclohexa-1,3-diene	204,4

36	14,319	5,73	Trans-β-Bergamotene	204,4
37	14,653	3,51	β-Curcumene	202,4
38	14,954	4,13	3-(1,5-Dimethyl-4-hexenyl)-6-methylene-cyclohexene [S-(RS)]	204,4
39	15,192	0,76	2-Naphthalenemethanol, decahydro-α,α,4a-trimethyl-8-methylene-, [2R-(2α,4α,8β)]	222,4
40	15,756	7,21	Nerolidol	222,4
41	16,097	1,54	β-Caryophyllene oxide	220,4
42	16,290	0,45	Guaiol	222,4
43	16,551	0,82	(1R,4R)-1-Methyl-4-(6-methylhept-5-en-2-yl)cyclohex-2-enol	210,35
44	16,945	1,74	β-Acorenol	222,4
45	17,408	3,14	Dehydrosesquicineole	218,4
46	18,659	27,5	Dihydroedulan	220,4
47	19,429	0,35	Xanthorhizol	234,4
48	19,880	0,36	Ageratriol	238,3
49	20,308	0,43	Z-α-Trans-bergamotenol	220,4
50	21,747	0,26	α-Cubebene	204,4
51	22,665	0,41	Copaene	204,4
52	23,162	0,24	3E-Muurola-3,5-diene	204,4
53	23,563	0,40	Biflora-4,10(19),15-triene	272,5
54	24,015	0,92	Biflora-4,10(19),15-trieno	272,5
55	24,829	2,39	Biflora-4(10),15-diene	272,5
56	25,415	0,03	Hexacosanal	380,7
57	26,025	0,52	(6Z,10Z)-Geranyl linalool	290,5
58	26,193	0,71	Geranyl-α-Terpinene	272,4
59	26,903	2,46	Phytol	296,5
60	27,615	0,09	all-trans-13,14-Dihydro Retinol	286,5
